# Buildings Contemplated

**Published:** 1914-06-13

**Authors:** 


### Buildings Contemplated.

Abingdon.—The Isolation Hospital Board have approved the plans for the county sanatorium to be built on a site adjoining the present isolation hospital.

Ashby-de-la-Zouch.—The Local Government Board have held an inquiry into an application of the Leicestershire County Council for sanction to a loan of £13,600 for the provision of an isolation hospital by the Ashby-de-la-Zouch Committee. The plans are by Mr. T. I. McCarthy, Coalville, Leicester.

Birmingham.—The Guardians are recommended to erect a new laundry at the Dudley Road Infirmary at an estimated cost of £18,400.

Canterbury.—The Kent County Asylums Committee invite tenders for the erection of a new bakery, new workrooms, additions to farm buildings, and other works in connection therewith at the Chartham Downs Mental Hospital. Specifications may be obtained of Mr. W. J. Jennings, architect, 4 St. Margaret's Street, Canterbury.

Chard.—The Guardians have agreed to build a hospital in the workhouse grounds, and to provide new laundry accommodation. The cost of the new infirmary will be about £3,000.

Chartham Downs.—The Kent County Asylums Committee invite tenders for the erection of a new bakery, new workrooms, additions to farm buildings, and other works at the County Mental Hospital at Chartham Downs, near Canterbury. The architect is Mr. W. J. Jennings, 4 St. Margaret's Street, Canterbury, from whom bills of quantities may be obtained.

Chester.—The City Council invite tenders for the erection of a tuberculosis ward, adjoining the isolation hospital, Sealand, Chester. Quantities may be obtained of the City Surveyor, Town Hall, Chester.

Chipping Ongar.—The Guardians invite tenders for the provision of additional bedroom accommodation and lavatories at the Children's Homes at Chipping Ongar, Essex. Specification and forms of contract may be obtained from Mr. Frank R. Coles, Clerk's Office, Hackney Union, Homerton, N.E.

Eastwood.—The Middlesex County Council have approved plans for a sanatorium at Eastwood at an estimated cost of £53,890.

Fareham.—The Hants County Council have passed estimates of £379 7s. 7d., £116 12s., and £674 4s. 4d. respectively for additions, staff cottages, and repairs at Fareham Mental Hospital.

Grassington.—The Bradford Corporation propose to build a sanatorium at Grassington at a cost of £30,270.

Howden.—The Rural District Council have accepted tenders amounting to £3,500 for a new isolation hospital.

Hill End.—Tenders are invited for erections of additions and alterations to the Hertfordshire County Mental Hospital, Hill End, St. Albans. Quantities and forms of tender may be obtained from the architects, Messrs. G. T. Hine and Pegg, 35 Parliament Street, Westminster, London, S.W.

Jersey.—The Jersey Dispensary and Infirmary is about to build a new institution with all modern requirements on a site in St. Helier, presented by Mr. C. G. Le Bas, who has also given £1,000 to the Building Fund. The committee has some £1,500 collected as a King Edward Memorial Fund at its disposal, and an anonymous donor has promised £500. The new institution is to have some thirty beds, including rooms for private patients; it is intended to start work in a few months' time.

Llanelly.—The Guardians have ordered the preparation of plans for a new hospital.

Sculcoates.—The House Committee recommend the Guardians to erect a building for 100 acute sick cases on the Grove House site.

Worksop.—The Guardians have decided to provide additional accommodation for children and the staff at a cost of £2,150.

